# Supplementary material for: Prognostic Significance of Baseline Blood Glucose Levels and Glucose Variability in Severe Acute Kidney Injury: A Secondary Analysis from the RENAL Study
Source: J Clin Med. 2022 Dec 20;12(1):15. doi: 10.3390/jcm12010015 (PMC9821032; doi:10.3390/jcm12010015)
Supplement: Supplementary file 1 [file jcm-12-00015-s001.zip › jcm-2087112-supplementary.pdf]

Supplementary Tables and Figures

Table S1c. Clinical characteristics of glucose variability

Characteristic	Group1 CV<16 (n=356)	Group2 16≤CV<22 (n=311)	Group3 22≤CV<30 (n=338)	Group4 CV≥30 (n=338)	*p* value
**Age (years)**	63.5 ± 15.5	62.4 ± 15.9	65.0 ± 14.6	66.4 ± 13.1	0.0003
**Male sex – no (%)**	235 (66.0)	208 (66.9)	208 (61.5)	215 (63.6)	0.28
**Time in ICU before randomisation (hour)**	70.2 ±174.4	44.7 ± 73.4	59.0 ± 129.2	35.0 ± 57.7	0.0007
**Mechanical ventilation – no. (%)**	260 (73.0)	235(75.6)	266(78.7)	236(69.8)	0.06
**Severe sepsis – no. (%)**	167(46.9)	170 (54.8)	164 (48.5)	167 (49.4)	0.86
**APACHE III score**	99.2 ± 25.5	100.1 ± 24.4	102.0 ± 24.1	106.8± 26.8	0.0005
**SOFA score**	10.1 ±2.8	10.6 ± 2.8	10.3 ± 2.6	10.3 ± 2.9	0.17
**Weight (kg)**	81.1 ±12.7	81.0 ± 13.3	80.8± 12.9	79.9 ± 12.6	0.57
**Albumin (g/L)**	26.8 ± 7.3	26.1 ±6.4	25.2 ±7.3	26.0 ±7.2	0.036
**Creatinine(ummol/L)**	360.3 ± 234.2	327.6 ± 194.6	321.7 ± 181.6	333.3 ± 215.6	0.07
**Potassium(mmol/L)**	4.8 ± 0.9	4.7 ± 0.8	4.8 ± 0.9	4.9 ± 1.0	0.10
**Phosphate (mmol/L)**	1.98 ± 0.76	1.96 ± 0.79	2.00 ± 0.81	2.06 ± 0.96	0.45
**Bicarbonate(mmol/L)**	19.2 ±6.1	18.8± 5.5	18.1 ± 5.7	17.8 ± 5.8	0.006
**No. of days in ICU**	**10.8± 12.9**	**15.1 ± 16.1**	**14.5 ± 15.8**	**9.8 ± 12.3**	**<0.0001**
**No. of days in hospital**	26.3± 26.0	30.3± 25.9	29.8 ± 24.7	22.1 ± 23.6	<0.0001
**Baseline glucose**	7.51± 2.45	7.41± 3.00	7.84± 3.05	8.89± 4.77	<0.0001

Table S2. Cox regression analysis on28-day mortality of baseline glucose level

Outcomes	Fourth of glucose		Event,n (%)	Univariate			multivariate		
	Category	n		HR（95%CI）	P value	P trend	HR（95%CI）	P value	P trend
**Death in 28 days**	<5.8	339	149(44.0)	1.58(1.20-1.98)	0.0006	0.37	1.47 (1.13-1.92)	0.042	0.09
	5.8-7.2	344	107(31.1)	1					
	7.2-9.1	362	136(37.6)	1.26(0.98-1.62)	0.076		1.25(0.96-1.63)	0.094	
	>9.1	360	137(38.1)	1.29(1.00-1.67)	0.047		1.09(0.83-1.42)	0.53	

Table S3. Cox regression analysis on28-day mortality of glucose variability CV

Outcome	Fourth of glucose CV（%）		Event,n（%）	HR（95%CI）	P Trend	multivariate	
	Category	n				HR（95%CI）	P trend
**Death in 28 days**	<16	356	119(33.4)	1	0.003	1	0.032
	16-22	311	87(28.0)	0.77(0.59-1.02)		0.70(0.52-0.94)	
	22-30	338	121(35.8)	0.99(0.77-1.28)		0.89(0.68-1.17)	
	>30	338	145(42.9)	1.37(1.07-1.74)		1.27(0.97-1.65)	

Table S4. Cox regression analysis on28-day mortality of SD

Outcome	Fourth of glucose SD		Event,n（%）	OR（95%CI）	P trend	multivariate	
	Category	n				OR（95%CI）	P value
**Death in 28 days**	<1.03	332	116(34.9)	1	0.18	1	0.09
	1.03-1.52	332	103(31.0)	0.81(0.62-1.06)		0.69(0.52-0.92)	
	1.52-2.21	344	118(34.3)	0.90(0.70-1.16)		0.84(0.63-1.11)	
	>2.21	335	135(40.3)	1.15(0.90-1.48)		1.26(0.94-1.69)	

Table S5. sensitivity analysis on90-day mortality of CV (7 days)

	Continuous glucose CV(%)	Quartile of CV（%）				P trend For Quartile
		Q1 <14	Q2 14-21	Q3 21-30	Q4 >30	
**n**	1340	319	335	332	354	
**Median of Glucose CV**	21	11	18	25	37	
**No of outcomes**	568	131	115	145	177	
**Crude**	1.01(1.01-1.02)	1	0.77(0.60-0.98)	1.03(0.82-1.31)	1.30(1.04-1.63)	0.0021
**Model 1**	1.01(1.01-1.02)	1	0.76(0.59-0.98)	1.03(0.81-1.31)	1.28(1.02-1.61)	0.0033
**Model 2**	1.01(1.01-1.02)	1	0.74(0.57-0.97)	0.99(0.77-1.27)	1.24 (0.98-1.58)	0.012
**Model 3**	1.02(1.01-1.03)	1	0.73(0.56-0.95)	1.00(0.78-1.29)	1.31(1.03-1.66)	0.0029

Table S6. sensitivity analysis on90-day mortality of SD (7 days)

	Continuous glucose STD	Quartile of STD				P trend For Quartile
		Q1 <0.98	Q2 0.98-1.45	Q3 1.45-2.18	Q4 >2.18	
**n**	1340	332	330	341	337	
**Median of Glucose CV**	1.45	0.71	1.20	1.78	2.79	
**No of outcomes**	568	141	133	137	157	
**Crude**	1.08(1.01-1.15)	1	0.88(0.68-1.14)	0.84(0.64-1.08)	1.14(0.89-1.46)	0.38
**Model 1**	1.08(1.01-1.15)	1	0.88(0.69-1.12)	0.87(0.69-1.11)	1.11(0.88-1.40)	0.38
**Model 2**	1.20(1.00-1.14)	1	0.81(0.63-1.04)	0.83(0.65-1.06)	1.04 (0.82-1.32)	0.93
**Model 3**	1.26(1.15-1.37)	1	0.84(0.65-1.07)	0.91(0.71-1.17)	1.33(1.03-1.72)	0.04

Table S7. Measures of variability for glucose variability measurements $x_1, x_2, \ldots x_n$

Measure		
Mean		$\bar{x} = \sum_{i=1}^{n} x_i / n$
SD	Sta7ndard deviation	$\sqrt{\frac{\sum_{i=1}^{n}(x_i - \bar{x})^2}{(n-1)}}$
CV	Coefficient of variation	100 x SD/ $\bar{x}$
